# Wind Tunnel Analysis of the Airflow through Insect-Proof Screens and Comparison of Their Effect When Installed in a Mediterranean Greenhouse

**DOI:** 10.3390/s16050690

**Published:** 2016-05-12

**Authors:** Alejandro López, Francisco D. Molina-Aiz, Diego L. Valera, Araceli Peña

**Affiliations:** Research Centre CIAIMBITAL, University of Almería, Ctra. Sacramento s/n, Almería 04120, Spain; alexlopez@ual.es (A.L.); fmolina@ual.es (F.D.M.-A.); apfernan@ual.es (A.P.)

**Keywords:** insect-proof screen, aerodynamic, wind tunnel, greenhouse, microclimate

## Abstract

The present work studies the effect of three insect-proof screens with different geometrical and aerodynamic characteristics on the air velocity and temperature inside a Mediterranean multi-span greenhouse with three roof vents and without crops, divided into two independent sectors. First, the insect-proof screens were characterised geometrically by analysing digital images and testing in a low velocity wind tunnel. The wind tunnel tests gave screen discharge coefficient values of *C_d,φ_* of 0.207 for screen 1 (10 × 20 threads·cm^−2^; porosity *φ* = 35.0%), 0.151 for screen 2 (13 × 30 threads·cm^−2^; *φ* = 26.3%) and 0.325 for screen 3 (10 × 20 threads·cm^−2^; porosity *φ* = 36.0%), at an air velocity of 0.25 m·s^−1^. Secondly, when screens were installed in the greenhouse, we observed a statistical proportionality between the discharge coefficient at the openings and the air velocity *u_i_* measured in the centre of the greenhouse, *u_i_* = 0.856 *C_d_* + 0.062 (R^2^ = 0.68 and *p*-value = 0.012). The inside-outside temperature difference Δ*T_io_* diminishes when the inside velocity increases following the statistically significant relationship Δ*T_io_* = (−135.85 + 57.88/*u_i_*)^0.5^ (R^2^ = 0.85 and *p*-value = 0.0011). Different thread diameters and tension affects the screen thickness, and means that similar porosities may well be associated with very different aerodynamic characteristics. Screens must be characterised by a theoretical function *C_d,φ_* = [(2*eμ*/*K_p_ρ*)·(1/*u_s_*) + (2*eY/K_p_*^0.5^)]^−0.5^ that relates the discharge coefficient of the screen *C_d,φ_* with the air velocity *u_s_*. This relationship depends on the three parameters that define the aerodynamic behaviour of porous medium: permeability *K_p_*, inertial factor *Y* and screen thickness *e* (and on air temperature that determine its density *ρ* and viscosity *μ*). However, for a determined temperature of air, the pressure drop-velocity relationship can be characterised only with two parameters: Δ*P* = *au_s_*^2^ + *bu_s_*.

## 1. Introduction

In a recent research work, Valera *et al.* [1] analysed the technological level of greenhouses in the province of Almería (Spain), observing that the main climate control system employed is natural ventilation, as 98.6% and 95.3% of the growers use side and roof vents, respectively. Only 4.2% of farmers use mechanical ventilation, 19.3% evaporative cooling systems and 8.4% heating systems. These data highlight the importance of natural ventilation in greenhouses in warm climates such as in the province of Almería. Natural ventilation is very important for optimal plant growth during the summer in Mediterranean countries [2]. For most of the year, a good natural ventilation system will allow growers to maintain suitable microclimate conditions inside the greenhouse for the crops [2,3,4]. However, Valera *et al.* [1] point out that the 14.4% average ventilation surface (ratio of total vent surface divided by total greenhouse surface, *S_V_*/*S_A_*) in Almería’s greenhouses is well below the minimum recommended value of 15%–30% [2,3,5,6,7,8].

In the province of Almería 99.1% of growers use insect-proof screens on the side vents and 95.4% have installed them on roof vents. In the case of the side vents, 58.3% of insect-proof screens are 15 × 30 threads·cm^−2^, while 25.6% are 10 × 20 threads·cm^−2^. The figures are similar for roof vents, 56.0% of which use screens of 15 × 30 threads·cm^−2^ as compared to 22.5% with 10 × 20 threads·cm^−2^ [1]. The porosity of these screens may vary considerably. Álvarez [9], for instance, analysed 14 commercial insect-proof screens of 10 × 20 threads·cm^−2^ and found that the thread diameter and the real density of the screens resulted in a porosity *φ* range of 31.1% to 40.3%. 

The use of insect-proof screens in greenhouses is considered essential to reduce the incidence of pests on the one hand [10,11] and on the other, to maintain those beneficial insects necessary for the crop inside the greenhouse [12]. Nevertheless, the screens clearly have a negative effect on the greenhouse microclimate by: (i) reducing the natural ventilation capacity [4,13,14,15]; and (ii) causing higher inside temperatures [3,4,16,17,18]. Although the negative effects of installing insect-proof screens have been studied previously, the results obtained by different authors vary greatly. Insect-proof screens with porosities from 25% to 53% were found to reduce the ventilation rate in comparison to a greenhouse without such screens between 77% and 16% [15,19,20,21,22,23,24,25].

Certain research works have analysed the aerodynamic characteristics of insect-proof screens and their influence on the natural ventilation and microclimate of greenhouses using Computer Fluid Dynamics (CFD) simulations [3,13,17,19,20,24,25,26,27]. The results obtained in these works vary significantly depending on whether the simulations were carried out on the real structure of the insect-proof screen or taking the screen as a porous structure [28]. Other works have analysed the natural ventilation of greenhouses using the gas tracer method [17,21,22,29,30,31,32,33,34]. However, depending on the positioning of the sensors, this technique may give rise to errors of up to 86% [35]. As a result, the literature presents varied and often conflicting results describing the influence of insect-proof screens on greenhouse microclimate and only in two works were the studied screens installed simultaneously in real greenhouses [16,18].

This work aims to characterise the aerodynamic behaviour of three insect-proof screens with different geometrical characteristics and to quantify the effect on the air velocity and temperature inside an empty Mediterranean multi-span greenhouse with roof ventilation, under summer climatic conditions. At this moment of the year, outside temperatures reached their highest values and the low developed crop transplanted in the greenhouses does not contribute significantly to the air cooling by transpiration, being necessary to improve natural ventilation.

## 2. Materials and Methods

Three insect-proof screens were analysed—screens 1 and 3 (10 × 20 threads·cm^−2^) and screen 2 (13 × 30 threads·cm^−2^)—for their effect on the microclimate of a Mediterranean greenhouse: (i) the geometric characteristics of each screen were determined in the laboratory; (ii) the screens were tested in a low-velocity wind tunnel; (iii) the microclimate of an experimental greenhouse divided with an inside plastic wall into two independent sectors was analysed. The comparison of screens 1 and 2 was carried out over two days, installing screens in the three roof windows of each sector that made up the experimental greenhouse. On the third day the screens were removed and replaced with screen 3 in both sectors of the greenhouse. Two days of experiments were again carried out with the same screen in both sectors. This last data allows us to both verify the similarity of the sectors (when the same screen was installed) and compare this third screen with the two previous ones.

### 2.1. Geometric Characterisation of the Insect-Proof Screens

**Figure 1:**
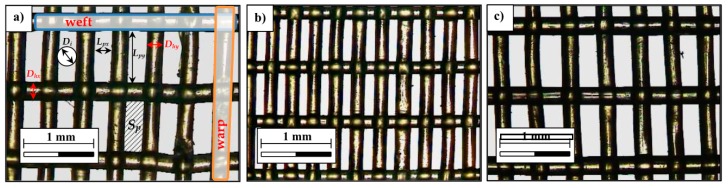
Geometric parameters determined using the methodology developed at University of Almería. Digital microscope images of screen 1 (**a**); screen 2 (**b**) and screen 3 (**c**).

The methodology used to characterise the geometry of the insect-proof screens was developed at the University of Almería [36,37,38]. For each insect-proof screen, three samples of approximately 2.04 cm^2^ were analysed. Twenty-four images were taken of each sample using a microscope fitted with a Motic DMWB1-223 digital camera (MoticSpain S.L., Barcelona, Spain) with a 4× lens and a resolution of 10.5 μm·pixels^−1^. Figure 1 presents the geometric parameters that were determined, as well as the porosity and density of the threads. For further details on the methodology, see Álvarez *et al.* [37]. The following geometric parameters were obtained: thread density *D_r_* (weft × warp) (threads·cm^−2^); porosity *φ* (%); weft pore length *L_px_* and warp pore length *L_py_* (µm); weft diameter *D_hx_* and warp diameter *D_hy_* (µm); mean thread diameter *D_h_* (µm); diameter of the circumference in the pore *D_i_* (µm); and pore surface area *S_p_* (mm^2^).

The thickness of the insect-proof screens was measured using the non-contact optical measurement equipment TESA-VISIO 300 (TESA SA, Renens, Switzerland) with a video camera of 0.05 μm resolution and a measurement uncertainty of (3 + 10·*e*/1000) (μm), where *e* is the measured dimension, which for the thickness of the screen resulted in a measurement uncertainty of less than 10 μm.

### 2.2. Aerodynamic Characterisation of the Insect-Proof Screens

The aerodynamic characteristics of the insect-proof screens were obtained using a low velocity wind tunnel, designed and developed at the University of Almería [4,39,40], with an auto-tuning PI automatic control system, using an open hardware and software platform [41]. In the wind tunnel, we have measured with two Pitot tubes and with a differential pressure transducer the pressure drop Δ*P* produced for different air velocities *u_s_* measured with a hot-film anemometer [41]. We have used a second-order polynomial Δ*P* = a*u_s_*^2^ + b*u_s_* + c [22,42] to fit the experimental data. This quadratic relationship is based on the theoretical Forchheimer equation [15] that uses two parameters to characterise the behaviour of a porous medium: screen permeability *K_p_* (m^2^) and the inertial factor *Y*. These parameters were calculated comparing the coefficients *a* and *b* of the experimental polynomial (zero order term *c* can be neglected compared with the other terms [42]) with the second- and first-order terms *a* and *b* of the and Forchheimer’s equation [40] and using the measured screen thickness *e* [40]. *K_p_* is a coefficient that depends on the geometry of the medium and is independent of the nature of the fluid. *Y* is a dimensionless form-drag constant dependent on the nature of the porous medium [15].

### 2.3. Experimental Greenhouse

**Figure 2:**
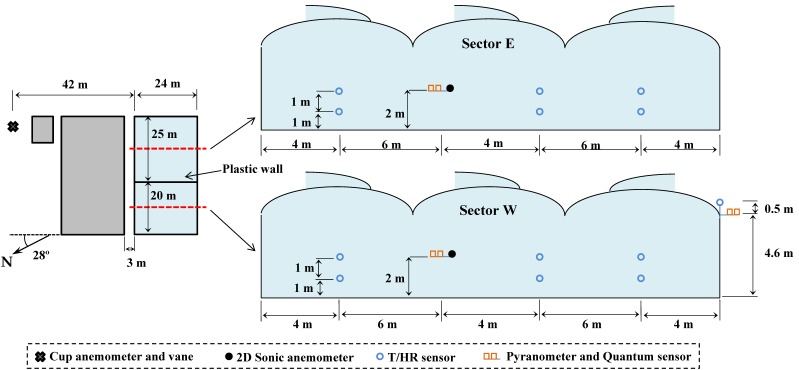
Location of the greenhouse sectors and layout of the climate sensors in the greenhouse.

The experiments were carried out in an empty multi-span Mediterranean greenhouse (24 × 45 m^2^) with three roof vents, located at the “Catedrático Eduardo Fernández” farm of the UAL-ANECOOP Foundation (36°51′ N, 2°16′ W) in the province of Almería. The greenhouse is permanently divided into two sectors by an interior plastic wall (Figure 2); sectors *E* (East) and *W* (West) measuring 24 × 25 m^2^ and 24 × 20 m^2^, respectively. The greenhouse is fitted with three roof vents measuring 40 × 1 m^2^ each (22.5 × 1 m^2^ in sector *E* and 17.5 × 1 m^2^ in sector *W*), with the same orientation to the wind in each sector. The ventilation surface, *i.e.*, surface area of the vent openings/greenhouse area, or *S_V_*/*S_A_*, was 11.25% for sector *E* and 10.81% for sector *W*.

Temperature and relative air humidity were measured inside and outside the greenhouse by means of 13 CS215 sensors (Campbell Scientific Spain S.L., Barcelona, Spain) with accuracy for temperature of ±0.4 °C over 5–40 °C and for relative humidity of ±2% over 10%–90% RH. The sensors were protected from radiation inside a naturally aspirated box 41003-5 (Campbell Scientific Spain S.L.). Air velocity was measured inside the greenhouse using 2 Windsonic bi-dimensional sonic anemometers (Gill Instrument LTD, Lymington, Hampshire, UK; resolution: 0.01 m·s^−1^; accuracy 2%). Solar radiation was measured inside and outside the greenhouse with three SP1110 pyranometers (Campbell Scientific Spain S.L.; sensitivity range of 350–1100 nm; accuracy of ±5%). The data from all sensors were stored in four CR3000 microloggers (Campbell Scientific Spain S.L.) with a frequency of 1 Hz. Outside wind speed was measured, at 10 m height, with a Meteostation II (Hortimax S.L., Almería, Spain), incorporating a cup anemometer (measurement range of 0 to 40 m·s^−1^; accuracy of ±5%) and a vane for wind direction (accuracy ±5°). The Meteostation II measurements were stored in an independent computer system once a minute. Figure 2 presents the location of the sensors in the experimental greenhouse.

Data were recorded on the 5th and 6th of August, 2014 with screen 1 (10 × 20 threads·cm^−2^) in the roof vents in sector *E* and screen 2 (13 × 30 threads·cm^−2^) in sector *W* of the greenhouse. On the 7th of August screen 3 (10 × 20 threads·cm^−2^) was installed in both sectors, and data were taken on both the 7th and 8th of August 2014. The vent openings were operated using a climate control system, with a setpoint temperature of 15 °C. Table 1 presents the means climatic conditions during the experiment.

### 2.4. Statistical Analysis

We have carried out regression analysis to compare the different variables for statistically significant differences (*p*-value < 0.05) conducted with Statgraphics Plus ver. 4.1 (Manugistics Inc., Rockville, MD, USA).

## 3. Results and Discussion

The first results presented concern the geometric characterisation of the insect-proof screens, highlighting the difference in porosity between screens 1 and 2. The second set of results was obtained in the low-velocity wind tunnel. That data obtained allow us to estimate the reduction in the greenhouse air renovation rate produced by the placement of the screens in the vent openings. It is expected that the reduction in the screen porosity will diminish the air renovation rate. The final section of the results analyses the microclimatic data obtained on the days of the experiments in the greenhouses.

### 3.1. Geometric Characteristics of the Insect-Proof Screens

The geometric analysis of the three insect-proof screens shows that number 3, installed in both sectors *E* and *W* for the second set of tests carried out in the experimental greenhouse, had the same thread density as screen 1 (10 × 20 threads·cm^−2^), but slightly higher porosity (Table 2). The main difference between screens 1 and 3 is the pore length *L_py_*. On the other hand, although screen 2 is less porous, its smaller pore size (weft pore length *L_px_*, warp pore length *L_py_*, diameter of the circumference drawn inside the pore *D_i_* and pore surface *S_p_*) constitutes an advantage as it will be more effective in preventing the access of harmful insects to the crop [12].

When choosing an insect-proof screen, therefore, growers must weigh two factors: (i) greater porosity will benefit the natural ventilation of the greenhouse, particularly in warmer climates; (ii) smaller pore size will benefit the insect-proof screen’s efficacy in preventing the entrance of pests in the greenhouse. Bethke and Paine [43] observed that insect-proof screens with pore size (*L_px_*) lower than twice the width of the insect’s thorax were effective in not allowing whitefly to pass through. Based on the geometric analysis (Table 2), screen 2 (*L_px_* = 110.0 ± 7.9 µm) is expected to be more efficient than screen 1 (*L_px_* = 238.6 ± 19.5 µm) and screen 3 (*L_px_* = 239.9 ± 18.5 µm) as a barrier against the whitefly *Bemisia tabaci*, with a mean thorax width of 215.8 µm and 261.8 µm for the male and female, respectively [43]. Álvarez [9] analysed the efficiency of several screens in preventing the entrance of *Bemisia tabaci*, concluding that insect-proof screens with mean *L_px_* values of between 187.3 μm and 250.3 μm were practically 100% effective. As a barrier against thrips (*Frankliniela occidentalis*), which has a mean thorax width of 184.4 µm and 245.5 µm for the male and female, respectively [43], screens 1 (*L_px_* = 238.6 ± 19.5 µm) and 3 (*L_px_* = 239.9 ± 18.5 µm) would not be effective, whereas screen 2 (*L_px_* = 110.0 ± 7.9 µm) would. However, one characteristic of this insect is that it can fold its wings, and it has been seen to be able to pass through screens with mean *L_px_* values of 118.5 µm [9]

### 3.2. Aerodynamic Characteristics of the Insect-Proof Screens

**Figure 3:**
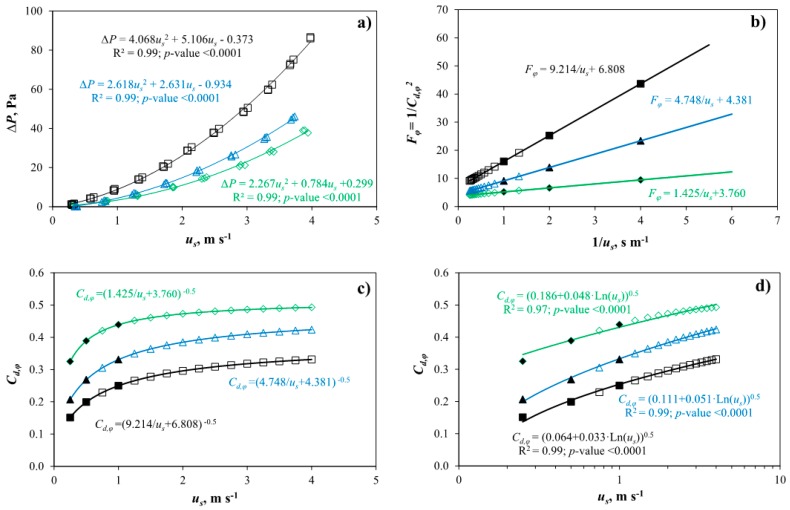
Pressure drops Δ*P* (Pa) measured in the wind tunnel experiments (**a**) and pressure drop coefficient due to the insect-proof screen *F_φ_* (**b**) in function of air velocity through screen *u_s_* (m·s^−1^) and its reciprocal 1/*u_s_*, respectively. Theoretical (**c**) and statistical (**d**) relationships between the discharge coefficient due to the insect-proof screen *C_d,φ_* and *u_s_*. Insect-proof screens 1 (Δ), 2 (□) and 3 (◊); values of *C_d,φ_* for air velocity equal to 0.25, 0.5 and 1.0 m·s^−1^ (▲, ■, ♦).

Table 3 presents the results obtained in the wind tunnel experiments at low velocities (0–4 m·s^−1^) for the three insect-proof screens (Figure 3a). Screen 2, with higher thread density (13 × 30 threads·cm^−2^) and lower porosity (26.3%) than the other two screens, gave rise to the highest pressure drop. Screen 1 with the same thread density (10 × 20 threads·cm^−2^) but with slightly lower porosity and greater thickness than screen 3 (Table 2) produces a greater pressure drop. Therefore, two insect-proof screens of the same thread density can have a different aerodynamic behaviour resulting from several minor geometric differences.

The parameter *K_p_* appears to increase with the screen porosity, which was also observed by Miguel *et al.* [42] and Teitel [16]. The latter work found that the inertial factor *Y* increased along with the porosity of the insect-proof screen, whereas the former work did not. In the present work, comparison of insect-proof screens 1 and 3 (35.0% and 36.0% porosity, respectively) showed that the inertial factor was greater for screen 3, which was less thick than screen 1.

On the other hand, screen 2, of less porosity and thickness than the other two, presented an intermediate recorded inertial factor. Therefore, the inertial factor seems to be related to the porosity and the thickness of the insect-proof screens. The aerodynamic characteristics of the insect-proof screens (*K_p_* e *Y*) allow us to determine the discharge coefficient due to the insect-proof screen *C_d,φ_* (Figure 3c).

The coefficient *C_d,φ_* can be calculated from the pressure drop coefficient *F_φ_* previously determined in the wind tunnel experiments (Figure 3b) [4,15]:

(1) $$C_{d,\phi }=\frac{1}{F_{\phi }^{0.5}}$$

(2) Fϕ=2eKp0.5(1Rep+Y)

In order to obtain a numerical value for *F_φ_*, we must calculate the Reynolds number based on the permeability of the insect-proof screen Re*_p_*. Applied to porous media, this Reynolds number can be calculated as [44]:

(3) Rep=Kpusρμ

where *u_s_* is the air velocity through the insect-proof screen, *ρ* is the air density (kg·m^−3^) and *μ* the air viscosity (kg·s^−1^·m^−1^) calculated for the daytime mean air temperature of 35 °C recorded in the experimental greenhouse. The physical relation between *C_d,φ_* and the velocity can be obtained from Equations (1)–(3):

(4) $$C_{d,\phi }={\left(\frac{1}{F_{\phi }}\right)}^{0.5}={\left(\frac{2e\mu }{K_{p}\rho }\frac{1}{u_{s}}+\frac{2eY}{K_{p}^{0.5}}\right)}^{-0.5}$$

In our case we have carried out this theoretical fit between the value of *C_d,φ_* and the air velocity through the screen *u_s_* (Figure 3c). This type of curve allows us to characterise the aerodynamic behaviour of each insect-proof screen, and it would be convenient if manufacturers were to provide this information. Others authors observed that the coefficient *C_d,φ_* was a function of the logarithm of the Reynolds number [16], which in turn depends on the air speed through the insect-proof screen *u_s_*. Thus, we can approach the theoretical function of *C_d,φ_* (Figure 3c) with a logarithmic relationship (Figure 3d), with a good correlation coefficient but based on a statistical fitting.

**Figure 4:**
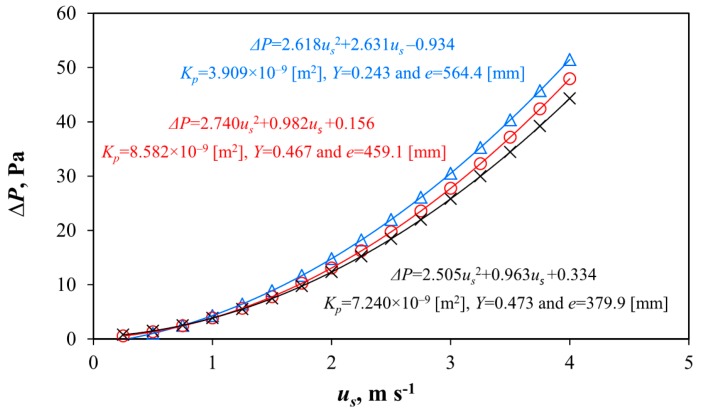
Pressure drops Δ*P* (Pa) in function of air velocity through screen *u_s_* (m·s^−1^), determined by the wind tunnel experiments for the insect-proof screen 1 (Δ) analyzed in this work, and for two screens with similar aerodynamic behavior but with different aerodynamic characteristics (*K_p_*, *Y* and *e*) and different ratio *a*/*b* (screens 5 and 9 in Valera *et al.*, 2006 [39] for low air velocities, 0–4 m·s^−1^).

We can characterise the aerodynamic behaviour of an insect-proof screen for a specific temperature of air during the test (influencing its density *ρ* and viscosity *μ*) with only two parameters, the coefficient *a* and *b* of the polynomial relationship between pressure drop and air velocity (Figure 3a). The ratio *a*/*b* defines the behaviour of screens at air velocities lower and greater than 1 m·s^−1^. For velocities lower than 1 m·s^−1^, the coefficient b is more important to compare two screens. However, for velocities greater than 1 m·s^−1^ the coefficient a become significate when analysing the pressure drop produced by the screens. To define the screens as a porous medium (with independence of the temperature during the test) we can use three parameters: the permeability *K_p_*, the inertial factor *Y* and the thickness *e*. These three parameters are used in CFD simulations of screens as porous media [3]. We can observe how there are screens with different values of *K_p_* and *Y*, but with different thickness resulting in similar different pressure drops (Figure 4).

In order to estimate the effect of the different insect-proof screens on the ventilation capacity of the experimental greenhouse, and therefore on the inside temperature, we have determined the total discharge coefficient *C_d_* for the roof vent of the experimental greenhouse. This coefficient can be calculated using the following expression [12,33,45]:

(5) $$C_{d}=\sqrt{\frac{1}{\frac{1}{C_{d,LH}^{2}}+\frac{1}{C_{d,\phi }^{2}}}}$$

The coefficient *C_d,LH_* depends on the geometry of the vent (equivalent to the coefficient *C_d_* of a vent without insect-proof screen) and can be obtained as follows [46]:

(6) $$C_{d,LH}={\left\{1.9+0.7\exp\left[-L_{V}/\left(32.5h\sin\alpha \right)\right]\right\}}^{-0.5}$$

where *L_V_* is the length of the vent (m), *h* the height of the vent (m) and *α* the angle of opening, which is 14° for the roof vent. For the roof vents in the experimental greenhouse *C_d,LH_* was 0.718 in sector *E* and 0.711 in sector *W*.

The discharge coefficient *C_d_* was calculated with insect-proof screen 1 on the roof vent in sector *E*, and with screens 2 and 3 on the roof vent in sector *W* (Table 4). It was determined for a reference air velocity us of 0.25, 0.5 and 1.0 m·s^−1^. The value of 0.25 m·s^−1^ for air velocity is the maximum mean value of the longitudinal component *u_x_*, perpendicular to the vents, observed in a similar experimental greenhouse [4].

The discharge coefficient values due to the presence of the insect-proof screen *C_d,φ_* presented in Table 4 were similar to those obtained in previous works following the same methodology [4,15]. The values of *C_d_* are similar to those obtained by other authors and compiled by Molina-Aiz *et al.* [15], in the range of 0.16 to 0.51 for vents with insect-proof screens whose porosity varied between 25% and 45%. For instance, in an Almería-type greenhouse fitted with an insect-proof screen of 34% porosity, a *C_d_* value of 0.194 was obtained [15]. When comparing *C_d_* values obtained by different authors, one should also bear in mind the fact that this coefficient depends on the Reynolds number Re*_p_* [15], which in turn depends on the air velocity through the porous medium.

The discharge coefficients obtained at the roof vents in each sector of the greenhouse allow us to estimate the reduction that one insect-proof screen will cause with respect to another one, based on the ratio *C*_*d,screen*2_/*C*_*d,screen*1_ [4,34]. Previous research works have used this ratio to compare greenhouses with and without insect-proof screens; for instance, Katsoulas *et al.* [21] and Kittas *et al.* [14] obtained a ratio of 0.67 for an insect-proof screen of 50% porosity and Kittas *et al.* [33] obtained a value of between 0.44 and 0.46 for an insect-proof screen of 60% porosity. In the present work the ratio *C_d,s_*_2_/*C_d,s_*_1_ was 0.74 (*u_s_* = 0.25 m·s^−1^), which indicates that in theory the less porous medium (screen 2) should bring about a 26% reduction in the natural ventilation capacity in sector *W* (with screen 2) of the greenhouse in comparison with sector *E* (with screen 1). Surprisingly, between the two commercial screens of similar thread density and porosity there is a similar difference in the discharge coefficient (*C_d,s_*_1_/*C_d,s_*_3_ = 0.67) to the one between the two commercial screens of different thread density (*C_d,s_*_2_/*C_d,s_*_1_ = 0.74). Wang *et al.* [47] also observed as two screens with the same bi-dimensional porosity and characteristic (length and width pore and filament width) produced different pressure drops, and therefore, different discharge coefficients, as consequence of its different tri-dimensional geometry.

After analysing screens 1, 2 and 3, we can suppose that the geometric characteristics of screen 2 make it more efficient as a barrier against insects, but we can state that its aerodynamic characteristics results in a greater reduction of the natural ventilation capacity of the greenhouse than those of screens 1 and 3.

### 3.3. Air Velocity Inside the Greenhouse

Between 12:00–16:00 on the 5th and 6th of August, the mean air velocity *u_i_* values on the horizontal plane XY were 0.16 m·s^−1^ in sector *W* (screen 2) and 0.27 m·s^−1^ in sector *E* (screen 1); which implies a 40.7% reduction in inside air velocity. On the 7th and 8th of August, with the same screen fitted in both sectors, the values of *u_i_* recorded were 0.28 m·s^−1^ in sector *W* and 0.34 m·s^−1^ in sector *E*; which implies a difference of 17.6%, which may be due to the difference in ventilation surface or the location of the anemometers. Kittas *et al.* [14] observed a 58% fall in the air velocity value inside a tunnel-type greenhouse (with only side vents) fitted with 50% porosity screens, in comparison to the greenhouse without screens.

**Figure 5:**
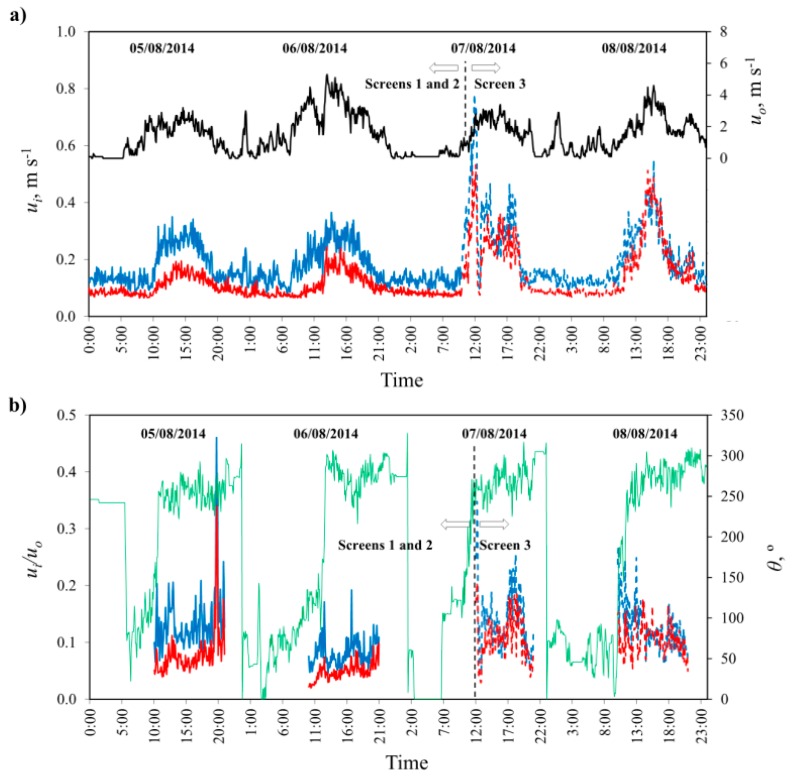
Temporal evolution of the outside wind speed *u_o_* (―) and inside air velocity *u_i_* (m·s^−1^) (**a**) and wind direction (―) and normalised velocity *u_i_*/*u_o_* (**b**). Inside velocities measured in the centre of sector *E* with screen 1 (―), sector *W* with screen 2 (―), sector *E* with screen 3 (- - -) and sector *W* with screen 3 (- - -).

In Figure 5 we can observe how the difference in air velocity at the centre of the two greenhouse sectors *u_i_* is more uniform at night, when the wind speed is low and the ventilation airflow is less notable, observing very similar differences for the four nights analysed (around 0.05 m·s^−1^). However, the difference between air velocity measured at the centre of the two sectors that constituted the greenhouse, increases at midday on the first days (5 and 6) when the screens installed in the two sectors were different, but decreases at midday on the last two days (7 and 8) when the screens are the same in both sectors (screen 3).

The wind tunnel experiments allowed us to estimate using Equation (4) that the expected reduction in the air renewal rate in sector *W* (screen 2) with respect to sector *E* (screen 1) due to the difference in aerodynamic characteristics of the screens was 26% (*C_d,s_*_2_/*C_d,s_*_1_ = 0.74). In a previous work, López *et al.* [4] measured a 16% reduction in the greenhouse air renewal rate with a less porous screen (*φ* = 33.5%) compared to a more porous one (*φ* = 39.0%) in agreement with the theoretical reduction in the air renewal rate of 11% (*C*_*d,*33.5%_/*C*_*d,*39.0%_ =0.89). These previous results confirm that the ratio *C*_*d,s*2_/*C*_*d,s*1_ provides an approximate value of the reduction in the renovation rate by natural ventilation produced by screen 2 respect to screen 1. The difference in aerodynamics characteristics of the three screens produces differences in the discharge coefficients of the vent openings, and consequently in the ventilation airflow, thus affecting the air velocity and temperature distribution inside the greenhouse. When a more permeable screen was used (*i.e.*, one with a greater discharge coefficient *C_d_*) we perceived higher air velocity at the centre of the greenhouse sector, indicating a greater ventilation airflow that cools the greenhouse air.

Although, the statistical relationship that best fits the experimental data is with an exponential function, we have use a linear relationship (*u_i_* = 0.856 *C_d_* + 0.062; R^2^ = 0.68 and *p*-value = 0.012) between the air velocity inside the greenhouse *u_i_* and the discharge coefficient of the windows *C_d_*. This can be explained by the direct relationship between the volumetric airflow *G* in the greenhouse and the discharge coefficient *C_d_* that multiply a function of inside-outside temperature difference in the greenhouse Δ*T_io_* and the wind speed *u_o_* [48]:

(7) $$G=C_{d}\cdot f\left(\Delta T_{io},u_{o}\right)+G_{o}\;[m^{3}s^{-1}]$$

where *G_o_* is the leakage airflow when openings are closed (m^3^·s^−1^).

The mean interior air velocity *u_i_* can be considered to be proportional to the ventilation volumetric flux *G* (Equation (7)) divided by the vertical cross-section surface perpendicular to the average direction of the inside airflow *S_CS_* [49]:

(8) $$u_{i}=\frac{G}{S_{CS}}\;[{\text{m s}}^{-1}]$$

We can deduce a linear relationship between the inside air velocity and the discharge coefficient of the vent openings:

(9) $$u_{i}=C_{d}\cdot S_{CS}\cdot f\left(\Delta T_{io},u_{o}\right)+S_{CS}\cdot G_{o}\;[{\text{m s}}^{-1}]$$

### 3.4. Air Temperature Inside the Greenhouse

**Figure 6:**
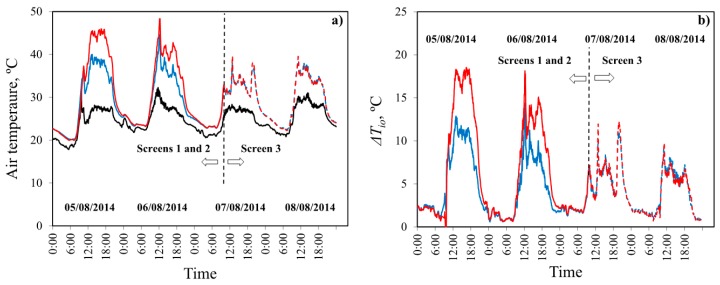
Air temperature (°C) (**a**) and inside-outside air temperature difference *∆T_io_* (°C) (**b**) on the days of the experiment. Outside (―); mean values of all the sensors in sector *E* with screen 1 (―), sector *W* with screen 2 (―), sector *E* with screen 3 (- - -) and sector *W* with screen 3 (- - -).

The first air temperature analysis compared the mean daily and daytime values and the mean values for the hottest part of day (12:00–16:00) in the two sectors of the greenhouse (Table 5). Using an insect-proof screen of 26.3% (screen 2) as opposed to 35.0% (screen 1) porosity gave rise to a mean daily temperature increase of 2.3 °C, while the increase in mean daytime temperature was 3.7 °C, and the increase during the hottest part of the day was 4.8 °C. The maximum temperatures recorded were 44.6 °C and 48.4 °C in sectors *E* and *W*, respectively, and the maximum difference between the two sectors at any given moment was 7.7 °C at the afternoon (Figure 6).

With insect-proof screen 3 in both greenhouse sectors (7th and 8th of August), no significant differences in temperature were observed (Figure 6), with mean values at midday of 34.9 °C and 34.8 °C in sectors *E* and *W*, respectively (Table 5). This indicates that the differences recorded on the 5th and 6th of August were primarily due to the different characteristics of screens 1 and 2. The mean temperatures recorded with insect-proof screen 3 fitted in both sectors of the greenhouse (7th and 8th of August), were lower than those recorded with screens 1 and 2 on the 5th and 6th of August (Table 5 and Figure 6), though outside temperatures were quite similar (Figure 6a). This ratifies that the better aerodynamic characteristics of screen 3 (higher *C_d_*) have improved the greenhouse ventilation capacity in comparison to screens 1 and 2, increasing the greenhouse’s cooling capacity and allow lower temperatures inside.

There is a proportional relationship between the thermal gradient values *∆T_io_* and the discharge coefficient *C_d_* [Δ*T_io_* = (−141.82 + 52.87/*C_d_*)^0.5^] and as consequence and the mean air velocity at the centre of the greenhouse *u_i_* [Δ*T_io_* = (−135.85 + 57.88/*u_i_*)^0.5^]. The difference in temperature between inside and outside is reduced by increasing *C_d_* and the air velocity. Teitel [16] also observed an increase of temperature and humidity inside the greenhouse when the pressure drop *F_φ_* increased, and therefore the discharge coefficient *C_d_* decreased. As mentioned in the introduction section, we have chosen to analyse the effect of insect-proof screen in the microclimate of empty greenhouses to avoid the cooling effect that plants transpiration produce in cropped greenhouses.

No differences were observed between the levels of inside absolute humidity *x_i_* between sectors, nor between inside *x_i_* and outside *x_o_* the greenhouse, Δ*x_io_* ≤ 0.0006 (kg·kg^−1^) (Table 5). This similarity in air humidity inside both greenhouse sectors, provide a guarantee that the main parameter that affects the inside greenhouse microclimate is the ventilation airflow affected by the type of insect-proof screens installed in the vent openings.

## 4. Conclusions

This research works provides new data on the effect that the aerodynamic characteristics of insect-proof screens have on the greenhouse microclimate. The thread density *D_r_* and porosity *φ* of the three screens tested were 9.8 × 20.0 threads·cm^−2^ and 35.0% (screen 1), 12.5 × 31.3 threads·cm^−2^ and 26.3% (screen 2) and 9.6 × 20.3 threads·cm^−2^ and 36.0% (screen 3).

The geometrical analyses of the three screens show how screen 3, with similar thread density and porosity to screen 1, has different thread diameter, pore length and thickness. These geometrical differences produce a change in the aerodynamic behaviour of the two screens in the wind tunnel tests. As a consequence of the different aerodynamic response of the three screens, we have obtained different values of discharge coefficients due to the presence of insect-proof screens *C_d,φ_* and of the roof vents *C_d_*. Thus, for an air velocity value of 0.25 m·s^−1^ (normally measured in the vent openings) we have obtained *C_d_* values of 0.199 for screen 1, 0.148 for screen 2 and 0.296 for screen 3.

When we measured the air velocity *u_i_* in the centre of the greenhouse, we observed that a more permeable screen (with a greater discharge coefficient *C_d_*) produces higher velocities, with statistical proportionality *u_i_* = 0.856 *C_d_* + 0.062; R^2^ = 0.68 and *p*-value = 0.012. The influence of the aerodynamic characteristics of the screens on airflow gives rise to variations in the inside temperature distribution. The inside-outside temperature difference *∆T_io_* is statistically correlated with the discharge coefficient Δ*T_io_* = (−141.82 + 52.87/*C_d_*)^0.5^ and with air velocity *∆T_io_* = (−135.85 + 57.88/*u_i_*)^0.5^ (R^2^ = 0.85 and *p*-value = 0.0011), and the temperature gradient diminishes when the inside air velocity increases.

Traditionally, screen porosity has been used as the characteristic parameter that determines their effect on the ventilation airflow and the greenhouse microclimate (temperature and humidity). However, the use of different thread diameters and tensions in the screens’ manufacture affects the screen thickness. This means that similar porosities may be associated with very different aerodynamic characteristics, and similar aerodynamic characteristics can be associated with very different pressure drop as consequence of the effect of different screen thickness. For this reason, we consider that our results indicate that screens need to be characterised by the theoretical function *C_d,φ_* = [(2*eμ*/*K_p_ρ*)·(1/*u_s_*) + (2*eY/K_p_*^0.5^)]^−0.5^ that relates the discharge coefficient of the screen *C_d,φ_* with the air velocity through the screen *u_s_*. This function can be calculated from the three parameters that characterise the pressure drop produced by each screen at the same air velocity: permeability *K_p_*, inertial factor *Y* and screen thickness *e*. However, for a determined temperature of air, the pressure drop-velocity relationship Δ*P* = *au_s_*^2^ + *bu_s_* can be characterised only with two parameters (coefficients *a* and *b*).

## Figures and Tables

**Table 1:** Daily climatic condition values: *u_o_*, outside wind velocity (m·s^−1^); *θ*, wind direction (°); *HR_o_*, outside relative air humidity (%); *T_o_*, outside air temperature (°C); *R_o_*, incoming shortwave radiation (W·m^−2^).

Date	*u_o_*	*θ* ^a^	*HR_o_*	*T_o_*	*R_o_*
5 August 2014	1.1 ± 1.0	219.5 ± 68.0	72.8 ± 16.6	23.7 ± 3.6	347.6 ± 404.4
6 August 2014	2.1 ± 1.4	179.6 ± 103.6	77.3 ± 10.0	25.4 ± 2.6	315.1 ± 389.4
7 August 2014	1.0 ± 1.0	240.2 ± 92.4	81.9 ± 11.2	24.3 ± 2.5	324.9 ± 390.8
8 August 2014	1.6 ± 1.0	179.8 ± 107.1	73.1 ± 14.6	25.5 ± 3.1	327.2 ± 395.3

^a^ Direction perpendicular to the vents is 208° for a wind from southwest (SW).

**Table 2:** Geometric characteristics of the insect-proof screens (Average value ± standard deviation): *D_r_*, thread density (threads·cm^−2^); *φ*, porosity (%); *L_px_*, weft pore length (µm); *L_py_*, warp pore length (µm); *D_hx_*, weft thread diameter (µm); *D_hy_*, warp thread diameter (µm); *D_h_*, mean thread diameter (µm); *D_i_*, diameter of the circumference drawn inside the pore (µm); *S_p_*, mean pore area (mm^2^).

Screen	*D_r_*	*φ*	*L_px_*	*L_py_*	*D_hx_*	*D_hy_*	*D_h_*	*D_i_*	*S_p_*
1	9.8 × 20.0	35.0	238.6 ± 19.5	746.0 ± 22.7	272.0 ± 7.2	261.2 ± 12.4	265.3 ± 11.9	241.7 ± 19.5	0.178 ± 0.015
2	12.5 × 31.3	26.3	110.0 ± 7.9	611.9 ± 17.5	187.7 ± 6.7	209.4 ± 8.2	200.2 ± 13.1	113.5 ± 8.5	0.067 ± 0.005
3	9.6 × 20.3	36.0	239.9 ± 18.5	765.4 ± 27.1	272.2 ± 11.6	252.0 ± 8.6	259.6 ± 13.9	241.9 ± 19.1	0.182 ± 0.015

**Table 3:** Aerodynamic characteristics of the insect-proof screens installed in the experimental greenhouse. *e*, thickness of the insect-proof screen (µm); a, b and c are the coefficients of the polynomial fit of the wind tunnel experiments; R^2^, coefficient of determination; *K_p_*, screen permeability (m^2^); *Y*, inertial factor; *F_φ_*, pressure drop coefficient that depends on the Reynolds number based on the permeability of the insect-proof screen Re*_p_*.

Screen	*e*	a	b	c	R^2^	*K_p_*	*Y*	*F_φ_*
1	564.4 ± 44.9	2.618	2.631	−0.934	0.99	3.909 × 10^−9^	0.243	18.05 (0.243 + Re*_p_*^−1^)
2	458.1 ± 44.2	4.068	5.106	−0.373	0.99	1.635 × 10^−9^	0.300	22.66 (0.300 + Re*_p_*^−1^)
3	508.1 ± 19.3	2.267	0.784	0.299	0.99	11.725 × 10^−9^	0.401	9.39 (0.401 + Re*_p_*^−1^)

**Table 4:** Discharge coefficients due to the presence of insect-proof screens *C_d,φ_* and of the roof vent *C_d_* (for *u_s_* of 0.25, 0.5 and 1.0 m·s^−1^). Insect-proof screen 1 (for sector *E* of the experimental greenhouse), insect-proof screen 2 (for sector *W*) and insect-proof screen 3 (for sector *W*). *φ*, porosity (%); *D_r_*, thread density (threads·cm^−2^).

Screen	*D_r_*	*φ*		*C_d,φ_*			*C_d_*	
			*u_s_* (m·s^−1^)					
			0.25	0.5	1.0	0.25	0.5	1.0
1	9.8 × 20.0	35.0	0.207	0.268	0.331	0.199	0.251	0.301
2	12.5 × 31.3	26.3	0.151	0.199	0.250	0.148	0.192	0.236
3	9.6 × 20.3	36.0	0.325	0.389	0.439	0.296	0.341	0.374

**Table 5:** Air temperature *T_i_* (°C) and absolute humidity *x_i_* (g·g^−1^) inside the greenhouse (average value ± standard deviation) and inside-outside temperature *∆T_io_* (°C) and absolute humidity *∆x_io_* (g·g^−1^). Subscripts: *E* sector east and *W* sector west.

	Daily		Daytime		Midday (12:00–16:00)		Midday (12:00–16:00)	
Date	*T_i,E_*	*T_i,W_*	*T_i,E_*	*T_i,W_*	*T_i,E_*	*T_i,W_*	*x_i,E_*	*x_i,W_*
	*Sector E with screen 1 (porosity 35.0%) and sector W with screen 2 (porosity 26.3%)*							
5 August 2014	29.3 ± 7.1	31.8 ± 9.8	34.1 ± 5.2	38.4 ± 7.6	38.2 ± 1.4	44.0 ± 1.8	0.0136 ± 0.0003	0.0142 ± 0.0004
6 August 2014	29.5 ± 5.7	31.6 ± 7.5	33.0 ± 5.0	36.3 ± 6.4	37.3 ± 2.6	41.2 ± 2.5	0.0157 ± 0.0004	0.0159 ± 0.0004
	*Sector E and W with screen 3 (porosity 36.0%)*							
7 August 2014	28.6 ± 4.6	28.8 ± 4.7	31.5 ± 3.6	31.7 ± 3.8	33.8 ± 1.5	34.0 ± 1.7	0.0148 ± 0.0005	0.0148 ± 0.0005
8 August 2014	29.0 ± 5.3	28.8 ± 5.3	32.4 ± 4.5	32.1 ± 4.5	36.0 ± 1.2	35.5 ± 1.1	0.0135 ± 0.0013	0.0134 ± 0.0013

## Nomenclature

*a*, *b*, *c*	second-order polynomial regression coefficients
*e*	thickness of the insect-proof screen (µm)
*h*	height of the vent openings (m)
*u_i_*	air velocity inside the greenhouse (m·s^−1^)
*u_o_*	outside wind velocity (m·s^−1^)
*u_s_*	air velocity through the screen (m·s^−1^)
*u_x_*	longitudinal component of air velocity (perpendicular to the vents)
*x*	absolute air humidity (g·g^−1^)
*C_d_*	total discharge coefficient of the opening
*C_d,LH_*	discharge coefficient due to the shape of the opening
*C_d,φ_*	discharge coefficient due to the presence of insect-proof screens
*D_h_*	diameter of the threads (µm)
*D_hx_*	diameter of the weft threads (µm)
*D_hy_*	diameter of the warp threads (µm)
*D_i_*	diameter of the inside circumference of the pore (µm)
*D_r_*	thread density (threads·cm^−2^)
*F_φ_*	pressure drop coefficient due to the presence of an insect-proof screen
*G*	volumetric ventilation flow (m^3^·s^−1^)
*G_o_*	leakage volumetric airflow when openings are closed (m^3^·s^−1^)
*HR_o_*	outside relative air humidity (%)
*K_p_*	screen permeability (m^2^)
*L_px_*	length of the pore in the weft direction (µm)
*L_py_*	length of the pore in the warp direction (µm)
*L_V_*	length of the vent openings (m)
*P*	Pressure (Pa)
R^2^	coefficient of determination
Re*_p_*	Reynolds number based on the insect-proof screen’s permeability
*R_o_*	incoming shortwave radiation (W·m^−2^)
*S*	sector
*S_A_*	greenhouse area (m^2^)
*S_CS_*	vertical cross-section surface perpendicular to the direction of the inside airflow (m^2^)
*S_p_*	area of the pore (mm^2^)
*S_V_*	surface area of the vent openings (m^2^)
*T*	air temperature (°C)
*Y*	inertial factor
*∆T_io_*	inside to outside temperature difference (°C)

## Greek Letters

*α*	angle of the vent opening (m)
*θ*	wind direction (°)
*μ*	air viscosity (kg·s^−1^·m^−1^)
*ρ*	air density (kg·m^−3^)
*φ*	porosity (%)
*∆*	difference (°C)

## Subscripts

*i*	inside the greenhouse
*o*	outside
*s*	screen
*E*	eastern sector
*W*	western sector
